# Prediction of walking ability following posterior decompression for lumbar spinal stenosis

**DOI:** 10.1007/s00586-021-06938-6

**Published:** 2021-08-05

**Authors:** Suzanne McIlroy, Feroz Jadhakhan, David Bell, Alison Rushton

**Affiliations:** 1 Physiotherapy Department, King’s College Hospital NHS Foundation Trust, Denmark Hill, London, SE5 9RS UK; 2 School of Population Health & Environmental Sciences, King’s College London, London, SE1 1UL UK; 3 Centre of Precision Rehabilitation for Spinal Pain [CPR Spine], School of Sport, Exercise and Rehabilitation Sciences, University of Birmingham, Birmingham, B15 2TT UK; 4 Neurosurgery Department, King’s College Hospital NHS Foundation Trust, Denmark Hill, London, SE5 9RS UK; 5 School of Physical Therapy, Western University, London, ON Canada

**Keywords:** Lumbar spinal stenosis, Walking, Outcome, Decompression, Prognosis

## Abstract

**Purpose:**

Following surgery for lumbar spinal stenosis (LSS) up to 40% of people report persistent walking disability. This study aimed to identify pre-operative factors that are predictive of walking ability post-surgery for LSS.

**Methods:**

An observational cohort study was conducted using data from the British Spine Registry (2017–2018) of adults (≥ 50 years) with LSS, who underwent ≤ 2 level posterior lumbar decompression. Patients receiving fixation or who had previous lumbar surgery were excluded. Walking ability was assessed by a single item on the Oswestry Disability Index and dichotomised into poor/good outcome. Multivariable regression models were performed.

**Results:**

14,485 patients were identified. Pre-operatively 30% patients reported poor walking ability, this decreased to 8% at 12 months follow-up. Predictors associated with poor walking ability at 12 months were: increasing age (≥ 75 years OR 1.54, 95% CI 1.07, 2.18), BMI ≥ 35 kg/m^2^ (OR 1.52, 95% CI 1.00, 2.30), severity of leg pain (OR 1.10, CI 95% 1.01, 1.21), disability (OR 1.01, 95% CI 1.01, 1.02) and quality of life (OR 0.72, 95% CI 0.56, 0.89). Pre-operative maximum walking distance (OR 1.10, 95% CI 1.05, 1.25) and higher education (OR 0.90, 95% CI 0.80, 0.96) were associated with reduced risk of poor walking ability at 12 months; *p* < 0.05. Depression, fear of movement and symptom duration were not associated with risk of poor outcome.

**Conclusion:**

Older age, obesity, greater pre-operative pain and disability and lower quality of life are associated with risk of poor walking ability post-operatively. Greater pre-operative walking and higher education are associated with reduced risk of poor walking ability post-operatively. Patients should be counselled on their risk of poor outcome and considered for rehabilitation so that walking and surgical outcomes may be optimised.

**Supplementary Information:**

The online version contains supplementary material available at 10.1007/s00586-021-06938-6.

## Introduction

Lumbar spinal stenosis (LSS) is a degenerative condition that occurs in approximately 10% of older adults, causing compression of nerves and blood vessels within the lumbar spine. LSS is characterised by neurogenic claudication: pain, numbness and sometimes weakness in the legs upon walking or standing [1]. Patients experience greater walking limitation than those with knee or hip osteoarthritis [2] and report a loss of sense of self and reduced participation in meaningful activities as a result of their functional limitations [3].

Initial management of LSS is typically physiotherapy and analgesia. However, if conservative management is unsuccessful, decompression surgery (most commonly posterior decompression) may be offered to reduce pain and improve function, specifically walking [4]. LSS is the most common indication for spinal surgery in older adults [1] with over 20,000 annual procedures performed in England annually [5]. There are considerable personal and healthcare costs associated with LSS [6] and this burden is expected to increase with the ageing population [1].

Post-surgical outcomes following LSS surgery are an area of debate. Whilst surgery decompresses the spinal nerves, the correlation between spinal canal size and walking ability is poor [7]. In addition, up to 40% of people report walking disability post-operatively [8] and, at six months post-surgery few achieve minimum physical activity recommendations [9]. These findings question decision-making processes for surgery. Knowledge of factors that are predictive of outcome following lumbar decompression surgery for LSS may be valuable to inform selection of patients, or expectation setting before surgery, and to inform rehabilitation post-operatively.

There are limited data on factors predictive of outcome following surgery for LSS. A systematic review published in 2006 (*n* = 21 studies, 7 at low risk of bias) found that being male, younger, having greater pre-operative walking ability, better self-reported health status and a higher income predicted better post-operative walking; whereas depression and cardiovascular comorbidities predicted poorer post-operative walking [10]. However, the authors were unable to calculate odds ratio or relative risks of the predictive factors due to the heterogeneity of the included studies. Therefore, the strength of the associations were not quantified, thus limiting confidence in findings to inform clinical decision-making. Subsequent studies have been at high risk of bias by excluding patients with missing data or under-powered with small sample sizes [11]. Therefore, a low risk of bias, adequately powered study is required. The use of surgical registries provides an opportunity to collect real-world data on large numbers of patients.

### Objective

To identify pre-operative factors that are predictive of walking ability at 6 weeks, 6 months and 12 months following posterior decompression for LSS.

## Methods

### Study design

An observational cohort study was conducted using data derived from the British Spine Registry (BSR) for all participants undergoing surgery in 2017 and 2018. Prior to surgery patients were invited to contribute to the registry; surgeons entered patient clinical, socio-demographic and surgical details and the patients were requested to complete self-reported outcome measures pre-operatively and at 6 weeks, 6 months and 12 months post-operatively. The outcome measures were completed on paper or electronically, either in clinic or via an email link. The *Strengthening the Observational Report on Epidemiology* (STROBE) guidelines [12] were used to inform design and reporting of the study. Ethical approval was obtained from the University of Birmingham Research Ethics Committee (ERN_19-1274AP1). Patients provided consent for their data to be used for evaluation purposes when they initially signed up to the registry.

### Data source

The BSR was launched in 2012 with the British Association of Spine Surgeons as the data controller. The registry allows all UK spinal surgeons to record information about patient diagnosis, co-morbidities, surgical procedures, complications; patient-reported outcome measures (PROMs) and patient-reported experience measures. The BSR aims to be a ‘whole practice’ registry covering lumbar degenerative, cervical degenerative, deformity, tumour, trauma, infection and intradural problems. Data for this study were extracted from the registry’s lumbar degenerative pathway.

### Participants

Adult patients aged ≥ 50 years old, entered in the BSR with a defined episode of 1 or 2 level lumbar posterior decompression surgery due to LSS (laminotomy, hemi-laminectomy, laminectomy, undercutting and/or partial facet joint resection) were included. Patients who received any form of fixation, micro-discectomy in isolation, surgery for non-degenerative cause (e.g. fracture, malignancy or infection) or who had had previous lumbar surgery were excluded.

### Candidate predictor variables

Potential predictors were all clinical and demographic details and PROMs collected pre-operatively: age, gender, comorbidities, body mass index (BMI), smoking status, education level (up to and including secondary school education: higher education), duration of symptoms, analgesia use, self-reported maximum walking distance and time able to stand, and working status. PROMs were used to assess back related disability (Oswestry Disability Index, ODI [13]), back and leg pain severity (Numerical rating scale, NRS), quality of life (EuroQuol five dimension, 5-level questionnaire [14], EQ5D), fear avoidance beliefs (Fear Avoidance Belief Questionnaire [15]) and depression (Zung depression questionnaire [16]).

### Outcome

Walking ability was assessed using a single item on the ODI [13]. The ODI is a widely used, validated, self-reported functional outcome measure specifically for use with people with low back pain. The ODI contains a single item asking patients to rate their walking ability from six statements ranging from “pain does not prevent me from walking” to “I am in bed most of the time”. It has been found to be a valid measure for self-rated walking ability [17]. To the best of our knowledge there is no published data on minimal clinical important difference for the single item. Therefore, walking ability was dichotomised into poor/good outcome based on patient’s response (Table 1). The dichotomy was defined a priori and based upon clinical judgment and the minimum clinically important difference of the ODI [18].

**Table 1:** Oswestry disability index walking item

ODI walking item responses	Score	Outcome
Pain does not prevent me walking any distance	0	Good outcome
Pain prevents me walking more than one mile	1	
Pain prevents me walking more than quarter of a mile	2	
Pain prevents me walking more than 100 yards	3	Poor outcome
I can only walk using a stick or crutch	4	
I am in bed most of the time and have to crawl to the toilet	5	

Table 1 demonstrates how walking outcome was dichotomised into good and poor outcome

ODI: Oswestry Disability Index

### Management of data

The effect of missing data for variables (e.g. BMI, smoking and PROMS) was dealt with by using multiple imputation using the Multivariable Imputation by Chained Equations (MICE) [19]. This technique replaces missing data with plausible values to estimate a more realistic regression coefficient, which means that variables with missing data are imputed one by one. Data within registries is also subject to data entry errors. To counteract this, extreme values were excluded.

### Statistical analysis

#### Descriptive statistics

All statistical analyses were conducted using STATA version 13.1 (Stata Corp, College Station Texas, USA). Descriptive statistics were used to summarise participants’ characteristics: means, standard deviation, medians and interquartile ranges (IQR) for continuous variables; and frequencies for categorical variables. Variability of distribution for each variable was tested separately. For data with high skewness the distribution was tested using histograms, with medians and IQR used to describe the central tendency and variability of the data.

#### Statistical modelling

To explore the influence of each predictive factor on poor outcome both linear and logistic multivariable regression models were fitted and mean differences or ORs including their 95% CIs for each candidate predictive factor reported. Multivariate analysis initially included all candidate predictive factors, and full results were reported. Selection of items for the model included those factors which were statistically significantly (*p* < 0.05) associated with poor outcome according to the univariate analysis and those deemed clinically relevant to retain.

## Results

The study population consisted of 14,485 adult patients aged ≥ 50 years following posterior decompression for LSS. Mean age of the study population was 68 ± 10.5 years and 51% were female. The mean pre-operative maximum distance able to walk was 167.3 m (± 226.3). Table 2 provides the clinical-demographic details and pre-operative PROMS for the study population.

**Table 2:** Baseline characteristics of the LSS (posterior decompression) population [*n* = 14,485]

Variable	Frequency	Percentage (%)
Age (years), mean (SD)	68.0 ± (10.5)	
Age categories		
< 60	3824	26.4
60–64.5	1912	13.2
65–69.5	1866	12.9
70–74.5	2433	16.8
≥ 75	4450	30.7
Gender		
Female	7353	50.8
BMI (kg/m^2^), median [IQR]	28.0 [25.1, 31.5]	
BMI (kg/m^2^), categories		
< 20	155	1.1
20–24.9	949	6.5
25–29.9	1828	12.6
30–34.9	1085	7.5
35–39.9	390	2.7
≥ 40	134	0.9
Missing	9944	68.2
Time stood (minutes), mean (SD)	9.8 (± 9.7)	
Missing	13,863	95.7
Distance walked (meters), mean (SD)	167.3 (± 226.3)	
Missing	13,776	95.1
Work status		
Yes	514	3.5
No	977	6.7
Missing	12,994	89.7
Education		
Less than secondary education	74	0.5
Post graduate degree	123	0.8
Secondary education	557	3.8
Some higher education	517	3.6
Undergraduate degree	212	1.5
Missing	13,002	89.8
Comorbidity		
Yes	2535	17.5
Cardiovascular disease	70	0.5
Diabetes	708	4.5
Inflammatory arthropathy	13	0.09
Ischaemic Heart Disease	418	2.9
Malignancy	40	0.3
Obesity	1,085	7.5
Osteoporosis	30	0.2
Rheumatoid arthritis	93	0.6
Renal failure	78	0.5
No	6594	45.5
Missing	5356	36.9
Duration of symptoms (years), mean (SD)	6.1 (± 8.8)	
Missing	13,084	90.1
Neurological deficit		
Cauda Equina Syndrome	236	1.6
Complete spinal cord injury	1	0.01
Incomplete spinal cord injury	27	0.2
No neurological deficit	6754	46.6
Radicular	5028	34.7
Missing	2439	16.8
Analgesia intake		
Yes	1357	9.4
No	130	0.9
Missing	12,998	89.7
Surgical approach		
Anterior and posterior	44	0.3
Posterior	14,441	99.7
Surgery type included discectomy		
Discectomy	5358	36.99
No discectomy	9125	63.0
Missing	2	0.01
Zung depression, median [IQR]		
Baseline (*n* = 463, 3.2%)	45 [38, 52]	
Missing (*n* = 14,022, 96.8%)		
Fear avoidance work, median [IQR]		
Baseline (*n* = 1,532, 10.6%)	6.0 [0, 21]	
Missing (*n* = 12,953, 89.4%)		
Fear avoidance physical activity, median [IQR]		
Baseline (*n* = 1,532, 10.6%)	16 [12, 20﻿]	
Missing (*n* = 12,953, 89.4%)		
Back pain (NRS), median [IQR]		
Baseline (*n* = 9257, 63.9%)	7.0 [5.0, 8.1]	
Missing (5228, 36.1%)		
Leg pain (NRS), median [IQR]		
Baseline (*n* = 9254, *n* = 63.9%)	8.0 [6.0, 9.0]	
Missing (*n* = 5,231, 36.1%)		
Quality of life (EQ-5D-5L-Health/NRS), median [IQR]		
Baseline (*n* = 9,015, 62.2%)	59 [40, 75]	
Missing (*n* = 5,470, 37.8%)		
Quality of life (EQ-5D-5L), median [IQR]		
Baseline (*n* = 9,019, 62.3%)	0.4 [0.21, 0.58]	
Missing (*n* = 5,466, 37.7%)		
Disability (ODI), median [IQR]		
Baseline (n = 9,084, 62.7%)	49 [36, 62]	
Missing (*n* = 5,401, 37.3%)		

BMI: body mass index; DISH: diffuse idiopathic skeletal hyperstosis; LSS: lumbar spinal stenosis; NRS: numerical rating scale; ODI: Oswestry Disability Index, IQR: interquartile range

### Evaluation of the pre to post walking ability

**Fig. 1:**
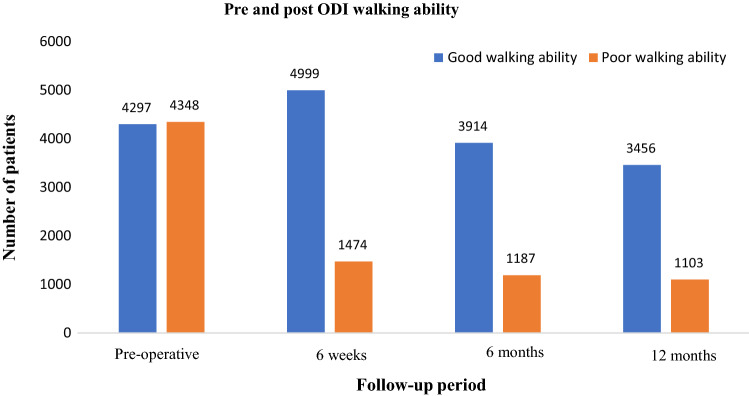
Walking ability: pre-operative, 6 weeks, 6- and 12-months follow-up

Table 3 and Fig. 1 illustrate the ODI walking ability at baseline and follow up. The number of patients with poor walking ability consistently decreased from baseline. At baseline 30% of patients (50% of participants with baseline score) reported poor walking ability, this decreased to 7.6% at 12 months follow-up (24.1% of participants with 12 month follow up outcome recorded). Post-operative poor outcome consistently decreased during follow-up period. Although a large number of patients had missing data during follow-up the ratio of good: poor walking improved post-operatively to six months followup, and then progress appeared to plateau.

**Table 3:** Walking ability as measured by the single item on the Oswestry Disability Index

Variable	Frequency	Percentage (%)
ODI (walking) scores – baseline		
Poor walking ability	4348	30.0
Good walking ability	4297	29.7
Missing	5840	40.3
ODI (walking) scores – 6 weeks		
Poor walking ability	1474	10.2
Good walking ability	4999	34.5
Missing	8012	55.3
ODI (walking) scores – 6 months		
Poor walking ability	1187	8.2
Good walking ability	3914	27.0
Missing	9384	64.8
ODI (walking) scores – 12 months		
Poor walking ability	1103	7.6
Good walking ability	3456	23.9
Missing	9926	68.5

Abbreviations: ODI: Oswestry Disability Index

### Determination of pre-operative factors that predict walking ability—univariate analysis

In the univariate analysis, severity of leg pain, quality of life, disability, and presence of a comorbidity were statistically significantly associated with risk of poor outcome (walking ability) (*p* =  < 0.05). The most common comorbidities were diabetes and obesity (Table 2). None of the other factors or PROMs showed statistically significant association with poor outcome (Table 4).

**Table 4:** Factors predicting walking ability 6 weeks, 6 and 12 months following surgery – univariate analysis

Pre-operative factors	6 week following surgery			6 months following surgery			12 months following surgery		
	Coefficient	95% CI of the coefficient	*P*-value	Coefficient	95% CI of the coefficient	*P*-value	Coefficient	95% CI of the coefficient	*P*-value
Age categories									
< 60	Ref			Ref			Ref		
60–64.5	0.02	(− 0.01, 0.05)	0.222	0.006	(− 0.05, 0.06)	0.803	− 0.007	(− 0.07, 0.06)	0.786
65–69.5	0.03	(− 0.03, 0.10)	0.272	− 0.01	(− 0.06, 0.04)	0.592	− 0.03	(− 0.12, 0.06)	0.489
70–74.5	0.03	(− 0.03, 0.09)	0.287	− 0.02	(− 0.09, 0.04)	0.372	− 0.05	(− 0.19, 0.08)	0.343
≥ 75	0.05	(− 0.04, 0.14)	0.195	− 0.006	(− 0.07, 0.06)	0.830	− 0.04	(− 0.22, 0.13)	0.527
Gender									
Female	Ref			Ref			Ref		
Male	− 0.02	(− 0.05, 0.01)	0.165	− 0.03	(− 0.09, 0.02)	0.177	0.01	(− 0.05, 0.08)	0.609
BMI Categories (kg/m^2^)									
< 20	Ref			Ref			Ref		
20–24.9	− 0.01	(− 0.12, 0.08)	0.744	− 0.02	(− 0.09, 0.05)	0.564	0.01	(− 0.07, 0.09)	0.792
25–29.9	− 0.001	(− 0.09, 0.09)	0.994	− 0.01	(− 0.09, 0.07)	0.783	0.02	(− 0.06, 0.10)	0.621
30–34.9	0.03	(− 0.06, 0.13)	0.508	0.02	(− 0.05, 0.10)	0.534	0.05	(− 0.03, 0.12)	0.209
35–39.9	0.07	(− 0.03, 0.18)	0.155	0.06	(− 0.008, 0.14)	0.084	0.09	(− 0.01, 0.19)	0.079
≥ 40	0.11	(− 0.01, 0.23)	0.123	0.07	(− 0,04, 0.18)	0.204	0.06	(− 0.05, 0.19)	0.253
Maximum walking distance (meters)	− 0.0001	(− 0.0002, 0.0001)	0.230	0.00003	(− 0.0003, 0.003)	0.807	0.0008	(− 0.003, 0.004)	0.626
Time stood (minutes)	0.001	(− 0.006, 0.009)	0.716	0.005	(− 0.001, 0.01)	0.086	0.003	(− 0.009, 0.01)	0.522
Neurological deficit No									
Yes	0.02	(− 0.04, 0.03)	0.852	0.003	(− 0.02, 0.03)	0.775	0.02	(− 0.03, 0.07)	0.446
Presence of comorbidity									
No	Ref			Ref			Ref		
Yes	− 0.07	(− 0.09, − 0.05)	**0.020**	0.07	(0.03, 0.10)	**0.007**	0.07	(0.02, 0.11)	**0.011**
Work status									
No	Ref			Ref			Ref		
Yes	− 0.07	(− 0.09, − 0.05)	**0.020**	0.08	(− 0.05, 0.21)	0.150	0.13	(− 0.05, 0.20)	0.126
Education									
Up to & including secondary	Ref			Ref			Ref		
Higher education	0.003	(− 0.03, 0.03)	0.818	0.005	(− 0.04, 0.21)	0.150	− 0.04	(− 0.01, 0.12)	0.231
Use of analgesia									
No	Ref			Ref			Ref		
Yes	− 0.009	(− 0.13, 0.11)	0.847	0.008	(− 0.17, 0.19)	0.907	− 0.13	(− 0.23, 0.09)	0.628
Surgery type									
No discectomy	Ref			Ref			Ref		
Discectomy	0.06	(0.01, 0.10)	**0.019**	0.12	(0.07, 0.17)	** < 0.001**	0.10	(0.06, 0.15)	** < 0.001**
Duration of symptoms (years)	0.003	(− 0.002, 0.009)	0.166	0.003	(− 0.001, 0.007)	0.121	0.004	(− 0.004, 0.007)	0.243
Zung depression score	0.006	(− 0.003, 0.003)	0.424	0.0007	(− 0.008, 0.009)	0.826	0.004	(− 0.01, 0.02)	0.561
Fear avoidance (work)	− 0.001	(− 0.006, 0.003)	0.434	− 0.002	(− 0.009, 0.004)	0.298	− 0.002	(− 0.01, 0.009)	0.638
Fear avoidance (pain)	− 0.0.006	(− 0.006, 0.005)	0.766	0.003	(− 0.002, 0.008)	0.186	0.002	(− 0.006, 0.01)	0.521
Back pain (NRS)	0.004	(− 0.0003, 0.009)	0.065	0.006	(− 0.004, 0.02)	0.172	0.005	(− 0.01, 0.02)	0.488
Leg pain (NRS)	− 0.01	(− 0.02, − 0.009)	** < 0.001**	− 0.01	(− 0.02, − 0.003)	**0.015**	− 0.009	(− 0.002, − 0.01)	**0.020**
Quality of life (EQ- 5D− 5L Health VAS)	0.00002	(− 0.001, 0.001)	0.953	− 0.0001	(− 0.0005, 0.0002)	0.343	0.0002	(− 0.0008, 0.001)	0.594
Quality of life (EQ-5D-5L)	− 0.07	(− 0.16, − 0.20)	**0.008**	− 0.09	(− 0.17, − 0.009)	**0.034**	− 0.05	(− 0.01, − 0.08)	**0.038**
Disability (ODI)	0.006	(0.005, 0.007)	** < 0.001**	0.008	(0.005, 0.01)	**0.003**	0.007	(0.003, 0.01)	**0.006**

Abbreviations: BMI: body mass index; CI: confidence interval; NRS: numerical rating scale; ODI: Oswestry Disability Index; VAS: visual analogue scale

Bold values represent *p* < 0.05

### Determination of pre-operative factors that predict walking ability—multivariate analysis

#### 6 weeks post-operative

In the multivariate analysis increasing age (except for 65–69.5 age group), BMI 35–39.9 kg/m^2^, level of education, severity of leg pain, quality of life, and back-related disability were predictive of poor post-operative walking ability (Table 5).

**Table 5:** Factors predicting walking ability 6 weeks, 6 months and 12 months following surgery – multivariate analysis

Pre-operative factors	6 week following surgery			6 months following surgery			12 months following surgery		
	OR	95% CI of the OR	*P*-value	OR	95% CI of the OR	*P*-value	OR	95% CI of the OR	*P*-value
Age categories									
< 60	Ref			Ref			Ref		
60–64.5	1.17	(1.03, 1.32)	**0.012**	1.08	(1.01, 1.24)	**0.011**	1.07	(1.01, 1.24)	**0.021**
65–69.5	1.24	(1.07, 1.45)	**0.004**	0.95	(0.82, 0.99)	**0.024**	1.17	(1.01, 1.19)	**0.042**
70–74.5	1.16	(0.97, 1.39)	0.095	0.88	(0.77, 1.39)	0.095	0.99	(0.76, 1.30)	0.986
≥ 75	1.62	(1.28, 2.04)	** < 0.001**	1.34	(1.11, 1.60)	**0.007**	1.54	(1.07, 2.18)	**0.024**
Gender									
Female	Ref			Ref			Ref		
Male	0.98	(0.89, 1.08)	0.733	0.97	(0.88, 1.07)	0.580	0.99	(0.88, 1.11)	0.947
BMI Categories (kg/m^2^)									
< 20	Ref			Ref			Ref		
20–24.9	0.91	(0.64, 1.31)	0.646	0.86	(0.60, 1.24)	0.442	1.06	(0.93, 1.55)	0.728
25–29.9	1.05	(0.74, 1.48)	0.770	0.94	(0.66, 1.33)	0.741	1.07	(0.75, 1.54)	0.685
30–34.9	1.19	(0.84, 1.71)	0.322	1.08	(0.75, 1.55)	0.656	1.25	(0.87, 1.82)	0.221
35–39.9	1.56	(1.05, 2.32)	**0.027**	1.24	(1.05, 1.87)	**0.015**	1.52	(1.00, 2.30)	**0.048**
≥ 40	1.39	(0.85, 2.29)	0.186	1.17	(1.02, 1.96)	**0.005**	1.34	(1.21, 5.23)	**0.015**
Maximum walking distance (meters)	0.99	(0.99, 1.00)	0.062	1.00	(1.02, 1.15)	**0.021**	1.10	(1.05, 1.25)	**0.027**
Time stood (minutes)	1.00	(0.98, 1.02)	0.711	1.01	(0.99, 1.02)	0.144	1.00	(0.98, 1.02)	0.501
Neurological deficit									
No	Ref			Ref			Ref		
Yes	1.05	(0.95, 1.15)	0.313	1.00	(0.92, 1.08)	0.958	0.96	(0.86, 1.07)	0.475
Presence of comorbidity									
No	Ref			Ref			Ref		
Yes	− 0.07	(− 0.09, − 0.05)	0.242	1.33	(0.98, 1.47)	0.564	1.37	(0.98, 1.67)	0.089
Work status									
No	Ref			Ref			Ref		
Yes	1.09	(0.81, 1.45)	0.486	1.21	(0.98, 1.47)	0.064	1.26	(0.97, 1.65)	0.072
Education									
Up to & including secondary school	Ref			Ref			Ref		
Higher education	0.79	(0.73, 0.86)	** < 0.001**	0.74	(0.67, 0.81)	** < 0.001**	0.90	(0.80, 0.96)	**0.005**
Use of analgesia									
No	Ref			Ref			Ref		
Yes	0.97	(0.71, 1.34)	0.856	0.97	(0.71, 1.34)	0.856	0.79	(0.54, 1.15)	0.190
Surgery type									
No discectomy	Ref			Ref			Ref		
Discectomy	1.07	(0.97, 1.18)	0.156	1.25	(1.11, 1.39)	** < 0.001**	1.14	(1.03, 1.28)	**0.013**
Durations of symptoms (years)	1.00	(0.99, 1.01)	0.082	1.00	(0.99, 1.01)	0.378	1.00	(0.99, 1.01)	0.233
Zung depression score	1.01	(0.99, 1.03)	0.171	1.00	(0.98, 1.01)	0.930	1.00	(0.97, 1.03)	0.512
Fear avoidance (work) (baseline)	0.99	(0.98, 1.01)	0.748	0.99	(0.97, 1.01)	0.412	0.99	(0.97, 1.03)	0.512
Fear avoidance (pain) (baseline)	0.99	(0.97, 1.02)	0.796	1.00	(0.99, 1.01)	0.136	1.00	(0.97, 1.01)	0.776
Back pain (NRS)	1.01	(0.98, 1.03)	0.335	1.03	(0.95, 1.12)	0.156	1.00	(0.98, 1.02)	0.868
Leg pain (NRS)	0.96	(0.93, 0.99)	**0.031**	1.01	(1.00, 1.04)	**0.007**	1.10	(1.01, 1.21)	**0.046**
Quality of life (EQ-5D-5L Health VAS)	1.00	(0.99, 1.00)	0.662	1.00	(0.99, 1.00)	0.271	1.00	(0.99, 1.00)	0.145
Quality of life (EQ-5D-5L)	0.70	(0.53, 0.93)	**0.016**	0.72	(0.53, 0.98)	**0.039**	0.72	(0.56, 0.89)	**0.037**
Disability (ODI)	1.01	(1.01, 1.02)	** < 0.001**	1.01	(1.01, 1.02)	** < 0.001**	1.01	(1.01, 1.02)	** < 0.001**

Abbreviations: BMI: body mass index; CI: confidence interval; VAS: visual analogue scale; ODI: Oswestry Disability Index; NRS: numerical rating scale

Bold values represent *p* < 0.05

#### 6 months post-operative

The results of the multivariate analysis identified that age, BMI 35–39.9 kg/m^2^, level of education, severity of leg pain, quality of life, and back-related disability continued to be predictive of poor post-operative walking ability. In addition, self-reported pre-operative maximum walking distance and BMI ≥ 40 kg/m^2^ were predictive of poor walking ability (Table 5).

#### 12 months post-operative

In the multivariate analyses, age, BMI ≥ 35 kg/m^2^, self-reported pre-operative maximum walking distance, level of education, severity of leg pain, quality of life, and back-related disability continued to be predictive of post-operative walking ability (Table 5).

## Discussion

We identified 14,485 patients registered on a national prospective registry receiving posterior decompressive surgery for LSS. Increasing age, BMI ≥ 35 kg/m^2^, self-reported pre-operative maximum walking distance, level of education, severity of leg pain, quality of life, and back-related disability were consistently associated with risk of poor walking ability six weeks to 12 months post-operatively. All other variables investigated were not found to be predictive.

Increasing age (except for the category 65–69.5 years) was found to be associated with risk of poor walking ability. Although this is consistent with the previous systematic review [10] subsequent studies have not demonstrated consistent results [20, 21]. The reason for the discrepancies are unclear, and may be due to methodological differences including difference in outcomes. When considering walking difficulty is associated with older age in the general population [22], older adults, especially ≥ 75 years should be considered to be at risk of poor walking ability post-operatively.

Following age ≥ 75 years, very high BMI (≥ 35 kg/m^2^) had the largest odds ratio for all the candidate variables investigated. This is consistent with observational studies investigating walking ability prior to surgery [23] and clinical outcomes post LSS surgery [24] thus adds credibility to our findings. As BMI is potentially modifiable, this result provides a possible target to address to improve walking ability post-surgery.

Pre-operative maximum walking distance (self-reported) was found to be associated with risk of poor walking ability post-operatively. This is consistent with the previous systematic reviews [10, 25] and provides credibility to our results. We also found disability and severity of leg pain to be significant factors however this is in contrast to a recent systematic review [25] who reported there was low evidence that disability and severity pain was not associated with walking capacity post-operatively. The reason for this discrepancy is unclear and is worth further investigation including the exploration of possible causation mechanisms.

Psychosocial variables have been identified as risk factors for walking disability in people with back pain [26] and other long term conditions [27, 28]. Within the current study the influence of fear avoidance, depression and quality of life was investigated. Only quality of life was found to be significantly associated with risk of poor post-operative walking ability, this is in contrast to a high quality study (*n* = 452) that reported no association between quality of life (measured by with the EQ5D-3L) and achieving the minimum clinical important difference in the function subscale of the Zurich Claudicant Questionnaire [29] at 12 months post-operative [21]. Our study is consistent with previous studies that did not identify an association between pre-operative fear of movement [30] or depression [31, 32] and post-operative walking in people with LSS. Interestingly in a group of 122 people undergoing surgery for LSS, mental health scores improved post-operatively [31]; and in further studies, continuous (i.e. present pre and post-operatively) fear of movement [30] and continuous depression [32] were demonstrated to be associated with post-operative walking ability whereas there was no association if present pre-operatively only. Thus it may be the post-operative psychosocial variables, rather than the pre-operative variables that are important in this cohort. This warrants further investigation as psychosocial factors are potentially modifiable and have been demonstrated to be a promising target for rehabilitation [33].

The current study has demonstrated that older age, BMI, pre-operative maximum walking distance, educational level, quality of life, leg pain and disability are associated with risk of poor walking post-operatively. Of these variables BMI, pre-operative walking distance, quality of life, pain and disability are potentially modifiable. Clinicians should therefore counsel their patients with these characteristics on their risk of poor outcome and encourage them to optimise their weight and health and consider referring them for rehabilitation. There have been promising results for pre-operative physiotherapy [34] and post-operative Cognitive-based Physical Therapy [33] to improve walking ability in adults with degenerative lumbar conditions. Future research may demonstrate it is possible to stratify patients at risk of poor outcome to receive rehabilitation, so that their post-operative outcomes and their walking are optimised.

### Strengths and limitations

Using a national surgical database, a large cohort of patients receiving surgery for LSS has been studied. The large number of participants included increases the generalisability of our results as participants do not represent one or two small clusters. Key variables predicting risk of poor walking ability post-operatively and areas that require further research have been identified.

There were large amounts of missing data. Missing data and loss to follow-up is an inherent problem with national databases. There is the concern that the missing data may be due to a systematic reason i.e. that it is not *missing completely at random* and this can introduce an inherent bias. In an attempt to reduce the risk of bias, and increase confidence in our results we included all registered patients [35] and clearly reported the missingness. However, there were large amounts of missing data thus selection bias cannot be completely ruled out. Additionally, there was a high loss to follow-up rate although, there remained a high number of participants at 12 months follow up (over 4500). In order to scrutinise our data and any impact from our imputations, we re-ran the analysis including participants with a completed ODI score at 12 months (see table 6 and 7, online supplementary information). This identified the same significant factors and thus increases the confidence, value, and generalisability of our results.

At baseline 30% of the included patients reported poor walking ability, this decreased post-operatively, with poor walking ability reported by 8.2% at 3 months follow-up and 7.6% at 12 months follow-up. This is a low prevalence compared to other reports [8] and may be due the impact of the missing data. Although the ODI is a well-recognised measure, we are not aware of other studies that have defined poor outcome in the same method as the current study. Our dichotomy was carefully considered to avoid a type I error however, future research should consider whether the dichotomy used to assess poor outcome is sufficiently sensitive. Due to the data retrieval process it was not possible to analyse the impact of individual comorbidities. This information could prove pertinent and is a recommended area for future research.

## Conclusion

In an analysis of over 14,000 patients from a national prospective spinal registry older age, higher BMI, greater severity of pre-operative leg pain and disability were associated with risk of poor walking ability and greater pre-operative maximum walking distance and higher education were associated with lower risk of poor walking ability six weeks to 12 months post-operative. Patients with these risk factors should be counselled on their risk of poor outcome and considered for rehabilitation so that walking and surgical outcomes may be optimised.

## Supplementary Information

Below is the link to the electronic supplementary material.

Supplementary file1 (PDF 186 KB)

## References

[1] Tomkins-Lane C, Melloh M, Lurie J (2016). ISSLS prize winner: consensus on the clinical diagnosis of lumbar spinal stenosis: results of an international delphi study. Spine (Phila Pa 1976).

[2] Winter CC, Brandes M, Müller C, Schubert T, Ringling M, Hillmann A, Rosenbaum D, Schulte TL (2010). Walking ability during daily life in patients with osteoarthritis of the knee or the hip and lumbar spinal stenosis: a cross sectional study. BMC Musculoskelet Disord.

[3] Lyle S, Williamson E, Darton F, Griffiths F, Lamb SE (2017). A qualitative study of older people's experience of living with neurogenic claudication to inform the development of a physiotherapy intervention. Disabil Rehabil.

[4] Ammendolia C, Stuber K, Tomkins-Lane C, Schneider M, Rampersaud YR, Furlan AD, Kennedy CA (2014). What interventions improve walking ability in neurogenic claudication with lumbar spinal stenosis? A systematic review. Eur Spine J.

[5] NHS Digital Hospital Admitted Patient Care Activity, 2017–18. 2018

[6] Rampersaud YR, Lewis SJ, Davey JR, Gandhi R, Mahomed NN (2014). Comparative outcomes and cost-utility after surgical treatment of focal lumbar spinal stenosis compared with osteoarthritis of the hip or knee–part 1: long-term change in health-related quality of life. Spine J.

[7] Kuittinen P, Sipola P, Saari T, Aalto TJ, Sinikallio S, Savolainen S, Kröger H, Turunen V, Leinonen V, Airaksinen O (2014). Visually assessed severity of lumbar spinal canal stenosis is paradoxically associated with leg pain and objective walking ability. BMC Musculoskelet Disord.

[8] Weinstein JN, Tosteson TD (2010). Surgical versus nonoperative treatment for lumbar spinal stenosis four-year results of the Spine Patient Outcomes Research Trial. Spine (Phila Pa 1976).

[9] Smuck M, Muaremi A, Zheng P, Norden J, Sinha A, Hu R, Tomkins-Lane C (2018). Objective measurement of function following lumbar spinal stenosis decompression reveals improved functional capacity with stagnant real-life physical activity. Spine J.

[10] Aalto TJ, Malmivaara A (2006). Preoperative predictors for postoperative clinical outcome in lumbar spinal stenosis: systematic review. Spine (Phila Pa 1976).

[11] Schulte TL, Schubert T (2010). Step activity monitoring in lumbar stenosis patients undergoing decompressive surgery. Eur Spine J.

[12] von Elm E, Altman DG, Egger M, Pocock SJ, Gøtzsche PC, Vandenbroucke JP, Initiative S (2007). The strengthening the reporting of observational studies in epidemiology (STROBE) statement: guidelines for reporting observational studies. Lancet.

[13] Fairbank JCT, Pynsent PB (2000). The oswestry disability index. Spine.

[14] Janssen MF, Pickard AS (2013). Measurement properties of the EQ-5D-5L compared to the EQ-5D-3L across eight patient groups: a multi-country study. Qual Life Res.

[15] Cleland JA, Fritz JM, Brennan GP (2008). Predictive validity of initial fear avoidance beliefs in patients with low back pain receiving physical therapy: is the FABQ a useful screening tool for identifying patients at risk for a poor recovery?. Eur Spine J.

[16] Zung WW (1965). A self-rating depession scale. Arch Gen Psychiatry.

[17] Anderson DB, Mathieson S, Eyles J, Maher CG, Van Gelder JM, Tomkins-Lane CC, Ammendolia C, Bella V, Ferreira ML (2019). Measurement properties of walking outcome measures for neurogenic claudication: a systematic review and meta analysis. Spine J.

[18] Asher AL (2018). Defining the minimum clinically important difference for grade I degenerative lumbar spondylolisthesis: insights from the Quality Outcomes Database. Neurosurg Focus.

[19] Azur MJ, Stuart EA, Frangakis C, Leaf PJ (2011). Multiple imputation by chained equations: what is it and how does it work?. Int J Methods Psychiatr Res.

[20] Sigmundsson FG, Kang XP, Jonsson B, Stromqvist B (2012). Prognostic factors in lumbar spinal stenosis surgery. Acta Orthop.

[21] Held U, Burgstaller JM (2018). Prognostic function to estimate the probability of meaningful clinical improvement after surgery - Results of a prospective multicenter observational cohort study on patients with lumbar spinal stenosis. PLoS ONE.

[22] Brach JS, Vanswearingen JM (2013). Interventions to improve walking in older adults. Curr Transl Geriatr Exp Gerontol Rep.

[23] Tomkins-Lane CC, Holz SC, Yamakawa KS, Phalke VV, Quint DJ, Miner J, Haig AJ (2012). Predictors of walking performance and walking capacity in people with lumbar spinal stenosis, low back pain, and asymptomatic controls. Arch Phys Med Rehabil.

[24] Pearson A, Lurie J, Tosteson T, Zhao WY, Abdu W, Weinstein JN (2012). Who should have surgery for spinal stenosis? Treatment effect predictors in SPORT. Spine.

[25] McIlroy S, Walsh E, Sothinathan C, Stovold E, Norwitz D, Norton S, Weinman J, Bearne L (2021) Pre-operative prognostic factors for walking capacity after surgery for lumbar spinal stenosis: a systematic review. Age Ageing. 10.1093/ageing/afab150 10.1093/ageing/afab150 34304266

[26] Hartvigsen J, Hancock MJ (2018). What low back pain is and why we need to pay attention. Lancet.

[27] Galea MN, Bray SR, Ginis KA (2008). Barriers and facilitators for walking in individuals with intermittent claudication. J Aging Phys Act.

[28] Danks KA, Pohlig RT, Roos M, Wright TR, Reisman DS (2016). Relationship between walking capacity, biopsychosocial factors, self-efficacy, and walking activity in persons poststroke. J Neurol Phys Ther.

[29] Stucki G, Liang MH, Fossel AH, Katz JN (1995). Relative responsiveness of condition-specific and generic health-status measures in degenerative lumbar spinal stenosis. J Clin Epidemiol.

[30] Burgstaller JM, Wertli MM, Steurer J, Kessels AG, Held U, Gramke HF, Group LS (2017). The Influence of Pre- and postoperative fear avoidance beliefs on postoperative pain and disability in patients with lumbar spinal stenosis: analysis of the lumbar spinal outcome study (LSOS) data. Spine.

[31] Kobayashi Y, Ogura Y (2019). The influence of preoperative mental health on clinical outcomes after laminectomy in patients with lumbar spinal stenosis. Clin Neurol Neurosurg.

[32] Sinikallio S, Aalto T, Airaksinen O, Lehto SM, Kroger H, Viinamaki H (2011). Depression is associated with a poorer outcome of lumbar spinal stenosis surgery: a two-year prospective follow-up study. Spine.

[33] Archer KR, Devin CJ (2016). Cognitive-behavioral-based physical therapy for patients with chronic pain undergoing lumbar spine surgery: a randomized controlled trial. J Pain.

[34] Fors M, Enthoven P, Abbott A, Oberg B (2019). Effects of pre-surgery physiotherapy on walking ability and lower extremity strength in patients with degenerative lumbar spine disorder: secondary outcomes of the PREPARE randomised controlled trial. BMC Musculoskelet Disord.

[35] Sterne JA, White IR, Carlin JB et al (2009) Multiple imputation for missing data in epidemiological and clinical research: potential and pitfalls. BMJ 338:b2393 10.1136/bmj.b2393 PMC2714692 19564179

