# Supplementary material for: Prediction of walking ability following posterior decompression for lumbar spinal stenosis
Source: Eur Spine J. 2021 Aug 5;30(11):3307–18. doi: 10.1007/s00586-021-06938-6 (PMC8550110; doi:10.1007/s00586-021-06938-6)
Supplement: Supplementary file 1 — Supplementary file1 (PDF 186 KB) [file 586_2021_6938_MOESM1_ESM.pdf]

Online supplementary information

Tables demonstrating univariate and multivariate analysis of factors predicting poor walking ability 12 months after surgery for data sets with 12 month ODI walking score (n=4,559)

Table 6: Factors predicting walking ability 6 months following surgery for complete data sets at 12 months – Univariate analysis

	**6 months following surgery**		
**Pre-operative factors**	**Coefficient**	**95% C of the coefficient**	***P*-value**
Age categories <60	Ref		
60-64.5	0.006	(-0.05, 0.06)	0.803
65-69.5	-0.01	(-0.06, 0.04)	0.592
70-74.5	-0.02	(-0.09, 0.04)	0.372
≥75	-0.006	(-0.07, 0.06)	0.830
Gender (female)	Ref		
Male	-0.03	(-0.09, 0.02)	0.177
BMI Categories (kg/m^2) <20	Ref		
20-24.9	-0.02	(-0.09, 0.05)	0.564
25-29.9	-0.01	(-0.09, 0.07)	0.783
30-34.9	0.02	(-0.05, 0.10)	0.534
35-39.9	0.06	(-0.008, 0.14)	0.084
≥40	0.07	(-0,04, 0.18)	0.204
Maximum walking distance (meters)	0.00003	(-0.0003, 0.003)	0.807
Time stood (minutes)	0.005	(-0.001, 0.01)	0.086
Duration of symptoms (years)	0.003	(-0.001, 0.007)	0.121
Neurological deficit No	Ref		
Yes	0.003	(-0.02, 0.03)	0.775
Presence of comorbidity No	Ref		
Yes	0.07	(0.03, 0.10)	**0.007**
Work status No	Ref		
Yes	0.08	(-0.05, 0.21)	0.150
Education Up to & including secondary school	Ref		
Higher education	0.005	(-0.04, 0.21)	0.150
Use of analgesia No	Ref		
Yes	0.008	(-0.17, 0.19)	0.907
Surgery type No discectomy	Ref		
Discectomy	0.12	(0.07, 0.17)	**<0.001**
Zung (baseline)	0.0007	(-0.008, 0.009)	0.826
Fear avoidance (work) (baseline)	-0.002	(-0.009, 0.004)	0.298
Fear avoidance (pain) (baseline)	0.003	(-0.002, 0.008)	0.186
Back pain (NRS)	0.006	(-0.004, 0.02)	0.172
Leg pain (NRS)	-0.01	(-0.02, -0.003)	**0.015**
Quality of life (EQ-5D-5L Health VAS)	-0.0001	(-0.0005, 0.0002)	0.343
Quality of life (EQ-5D-5L)	-0.09	(-0.17, -0.009)	**0.034**

	6 months following surgery		
**Pre-operative factors**	**Coefficient**	**95% C of the coefficient**	***P*-value**
Disability (ODI)	0.008	(0.005, 0.01)	**0.003**
Abbreviations: BMI: body mass index; VAS: visual analogue scale; ODI: Oswestry Disability Index; NRS: numerical rating scale			

Table 7: Factors predicting walking ability 6 months following surgery for complete data sets at 12 months – Multivariate analysis

	6 months following surgery		
**Pre-operative factors**	**OR**	**95% Confidence Interval**	***P*-value**
Age categories<br><60	Ref		
60-64.5	1.08	(1.01, 1.24)	**0.011**
65-69.5	0.95	(0.82, 0.99)	**0.024**
70-74.5	0.98	(0.97, 1.39)	0.095
≥75	1.34	(1.11, 1.60)	**0.007**
Gender<br>(female)	Ref		
Male	0.97	(0.88, 1.07)	0.580
BMI Categories (kg/m^2)<br><20	Ref		
20-24.9	0.86	(0.60, 1.24)	0.442
25-29.9	0.94	(0.66, 1.33)	0.741
30-34.9	1.08	(0.75, 1.55)	0.656
35-39.9	1.24	(105, 1.87)	**0.015**
≥40	1.17	(1.02, 1.96)	**0.005**
Maximum walking distance (meters)	1.10	(1.02, 1.15)	**0.021**
Time stood (minutes)	1.01	(0.99, 1.02)	0.144
Duration of symptoms (years)	1.00	(0.99, 1.01)	0.378
Neurological deficit<br>No	Ref		
Yes	1.00	(0.92, 1.08)	0.958
Presence of comorbidity<br>No	Ref		
Yes	1.33	(0.98, 1.47)	0.564
Work status<br>No	Ref		
Yes	1.21	(0.98, 1.47)	0.064
Education<br>Up to & including secondary school	Ref		
Higher education	0.74	(0.67, 0.81)	**<0.001**
Use of analgesia<br>No	Ref		
Yes	0.97	(0.71, 1.34)	0.856
Surgery type<br>No discectomy	Ref		
Discectomy	1.25	(1.11, 1.39)	**<0.001**
Zung (baseline)	1.00	(0.98, 1.01)	0.930
Fear avoidance (work) (baseline)	0.99	(0.97, 1.01)	0.412
Fear avoidance (pain) (baseline)	1.00	(0.99, 1.01)	0.136
Back pain (NRS)	1.03	(0.95, 1.12)	0.156
Leg pain (NRS)	1.01	(1.00, 1.04)	**0.007**
Quality of life (EQ-5D-5L Health VAS)	1.00	(0.99, 1.00)	0.271

Prediction of walking ability following posterior decompression for lumbar spinal stenosis

	**6 months following surgery**		
**Pre-operative factors**	**OR**	**95% Confidence Interval**	***P*-value**
Quality of life (EQ-5D-5L)	0.72	(0.53, 0.98)	**0.039**
Disability (ODI)	1.01	(1.01, 1.02)	**<0.001**
Abbreviations: BMI: body mass index; VAS: visual analogue scale; ODI: Oswestry Disability Index; NRS: numerical rating scale			
